# Telomere Length and Regulatory Genes as Novel Stress Biomarkers and Their Diversities in Broiler Chickens (*Gallus gallus domesticus*) Subjected to Corticosterone Feeding

**DOI:** 10.3390/ani11102759

**Published:** 2021-09-22

**Authors:** Kazeem Ajasa Badmus, Zulkifli Idrus, Goh Yong Meng, Awis Qurni Sazili, Kamalludin Mamat-Hamidi

**Affiliations:** 1 Department of Animal Science, Universiti Putra Malaysia, Seri Kembangan 43400, Selangor, Malaysia; alqasiminternational@yahoo.com (K.A.B.); zulidrus@upm.edu.my (Z.I.); awis@upm.edu.my (A.Q.S.); 2 Institute of Tropical Agriculture and Food Security, Universiti Putra Malaysia, Seri Kembangan 43400, Selangor, Malaysia; ymgoh@upm.edu.my; 3 Department of Veterinary Pre-Clinical Science, Universiti Putra Malaysia, Seri Kembangan 43400, Selangor, Malaysia

**Keywords:** broiler, corticosterone, performance, telomeres, telomere regulators, stress biomarkers

## Abstract

**Simple Summary:**

Assessment of poultry welfare is very crucial for sustainable production in the tropics. There is a demand for alternatives to plasma corticosterone levels as they have received much criticism as an unsuitable predictor of animal welfare due to inconsistency. In this study, we noticed no effect of age on plasma corticosterone (CORT) although it was altered by CORT treatment. However, growth performances and organ weight were affected by CORT treatment and age. The broad sense evaluation of telomere length in this study revealed that telomere length in the blood, muscle, liver and heart was shortened by chronic stress induced by corticosterone administration. The expression profile of the telomere regulatory genes was altered by chronic stress. This study informed us of the potential of telomere length and its regulatory genes in the assessment of animal welfare in the poultry sector for sustainable production.

**Abstract:**

This study was designed to characterize telomere length and its regulatory genes and to evaluate their potential as well-being biomarkers. Chickens were fed a diet containing corticosterone (CORT) for 4 weeks and performances, organ weight, plasma CORT levels, telomere lengths and regulatory genes were measured and recorded. Body weights of CORT-fed chickens were significantly suppressed (*p* < 0.05), and organ weights and circulating CORT plasma levels (*p* < 0.05) were altered. Interaction effect of CORT and duration was significant (*p* < 0.05) on heart and liver telomere length. CORT significantly (*p* < 0.05) shortened the telomere length of the whole blood, muscle, liver and heart. The *TRF1*, *chTERT, TELO2* and *HSF1* were significantly (*p* < 0.05) upregulated in the liver and heart at week 4 although these genes and *TERRA* were downregulated in the muscles at weeks 2 and 4. Therefore, telomere lengths and their regulators are associated and diverse, so they can be used as novel biomarkers of stress in broiler chickens fed with CORT.

## 1. Introduction

Broilers are prone to many welfare issues related to genetic differences and environmental challenges. Monitoring animal genetics and physiological data play a pivotal role in the assessment of their welfare [1]. Thus, there is an increasing demand for reliable biomarkers to monitor the health and well-being of poultry in relation to different environmental stresses. The common conventional well-being biomarkers such as hematological values and plasma corticosterone (CORT) levels are not reliable due to inconsistencies in their results [1,2]. The chronic stressor, CORT, has been observed as the product of the hypothalamic–pituitary–adrenal axis response to stress in birds [3,4,5]. It generates reactive oxygen species (ROS), causing oxidative damage [6]. Many reports show that the elevation of CORT due to stress, be it from plasma or serum, is short-lived due to the biological clock phenomenon and it is known to revert to the normal level after some time [6,7]. However, telomere length and its regulatory genes such as telomerase (*chTERT*), telomeric repeat transcriptional factor 1 (*TRF1*), telomeric repeat-containing RNA (*TERRA*) and heat shock factor 1 (*HSF1*) have recently been shown to be consistently correlated with stress responses [8,9]. Telomeres are nucleoprotein (TTAGGG repeats) structures located at the ends of chromosomes and are usually eroded when exposed to stresses [10,11]. This could indicate the ability to cope with stressful situations, i.e., adaptations [12] and could predict longevity, reproductive capacity or fitness to survive in birds [13,14]. Telomeres are mainly used to safeguard chromosomes and to protect genomic stability by stopping continuous recombination of cells [15]. Several factors, including oxidative stress, feed restriction, antioxidant, breed, sex, age and stocking density could influence the rate of telomere shortening in chicken [16,17]. Chronic stress and reactive oxygen species (ROS) production induced due to CORT administration have been reported to cause drastic reductions in telomere lengths in wild birds [18,19]. In addition, uncovering the role of the sizes of organs on the telomeric DNA becomes important. Organs with high metabolic rate such as liver, heart, brain, and kidney have been reported to be higher in size at an early age [20]. Implication of increase in sizes of these organs is that high metabolic activities will be triggered and will consequently increase the ROS because of increased resting energy expenditure (REE). Metabolic REE was observed to be higher in children per kilogram body weight and reduces steadily during growth [20]. It has been observed that in the first year of life in humans, organs grow in proportion to body weight, but later organ growth decelerates [21]. Decline in the proportional weight of metabolically active organs results in decrease in REE [22]. Association of organ sizes with telomere length can be a good stress indicator for animal welfare assessment.

Telomere length maintenance and restoration due to stress can be assessed via the expression level of the regulatory genes. Shelterin complex that consists of several subunit proteins is crucial for telomere maintenance and genome integrity. A component of shelterin complex, *TRF1*, facilitates and supports the recovery of the telomeric DNA during T-loop formation [23,24,25]. In addition, telomerase remains essential in telomere integrity as it attaches nucleotides synthesized from the shelterin genes to the telomeric end to maintain the telomere [26,27]. Telomere lengths were maintained in cancer and stem cells of the germline that expressed high levels of telomerase. However, reduced amounts of telomerase were reported in the somatic cell during stress, leading to progressive shortening of the telomere length [28,29]. The telomere maintenance gene 2 (*TELO2*) is another gene that exerts its action via telomerase. Though its expression in chicken fed with CORT is not yet known, it has been predicted to perform a vital role in the telomeric DNA-binding activity [30]. Furthermore, the RNA molecule called telomeric repeat-containing RNA (*TERRA*) was noted as ensuring that very short (or damaged) telomeres are regenerated in humans [31]. This mechanism allows *TERRA* to repair eroded telomeres so that cells can continue to live and keep regenerating. How *HSF1* controls telomerase is unknown to us but it has been reported that human fibroblast showing deficiency in *HSF1* experienced telomeric DNA damage [32]. This shows that *HSF1* is vital for *TERRA* and hence telomerase elevation in a cell under influence of stress. Several studies have recently reported on the role of heat shock in stimulating the upsurge of *TERRA* [33,34]. 

The reports on how plasma CORT level could be used to predict animal physiology and how it affects traits of economic importance in chicken have been widely criticized [1,7], thus necessitating the current study. We intended to test the hypothesis that CORT administration elevated circulating plasma CORT level and altered telomere length. This study was therefore designed to examine the effects of CORT as a stressor on telomere length, its association with the regulatory genes and how they could be used as novel stress biomarkers in broiler chickens fed with CORT.

## 2. Materials and Methods

### 2.1. Animal Management, Housing and Experimental Design

This study was conducted in compliance with the Animal Utilization Protocol approved by the Institutional Animal Care and Use Committee (IACUC) of Universiti Putra Malaysia (approval number: UPM/IACUC/AUP-R019/2018). A total of one hundred (100) male day-old Cobb500 (Leong Hup Farm, Kuala Lumpur, Malaysia) chicks were used for this study and were subjected to 2 × 2 completely randomized factorial design. Chicks were randomly assigned by treatment in groups of 10 into 10 battery cages and subsequently weighed and wing banded. All the cages were placed in a single environmentally controlled chamber. The area of the cages measured 122 cm in length, 91 cm in width and 61 cm in height. The experiment began with initial temperature fixed at 32 °C on day 1 and gradually lowered until it reached day 21. Relative humidity ranged from 70% to 80%. The chickens were vaccinated with infectious bronchitis disease virus and Newcastle disease virus vaccines at day 7 and 14, respectively.

### 2.2. Diets and Corticosterone Challenge

The diets for the experiment consisted of starter (crumble form) and finisher (pellet form) which were provided ad libitum. The crude protein (CP) of the diet was 21.0% in the starter phase and 19.0% in the finisher phase. Chickens were raised for the first 14 days without CORT feeding (week 2) and then subjected to 30 mg/kg diet CORT feeding [35] on day 15 of age (beginning of week 3) for another 28 days (week 6). The control group consisted of 50 chickens that were given a commercial diet free from CORT (Table 1). The CORT (Abcam, Cambridge, UK) used was an endogenous steroid hormone with an apoptotic-inducing property.

Five hundred mg of CORT was dissolved in 20 mL ethanol for proper solubility before mixing with feed. The CORT mixture was then thoroughly mixed with 16.67 kg feed, producing 30 mg CORT per one kilogram feed. Mixings were repeated at intervals as the diets were being consumed throughout the experiment. Both the CORT and the control group consisted of 5 replicates with 10 chickens each in a cage. Only one level of CORT feeding was used in this study [35].

### 2.3. Growth Rate Parameters and Animal Sampling

Body weights and feed intakes (FI) of all the chickens were measured weekly using a weighing scale (CAS Corporation, Seoul, Korea). The FI was adjusted for mortality which was registered upon occurrence. The feed conversion ratio (FCR) was calculated as feed/body weight gain. At the end of week 4 and 6 of age, equivalent to 2 and 4 weeks of CORT administration, two (2) chickens were sampled at random from each cage from CORT and control groups for slaughtering. The slaughtering was humanely performed by severing the jugular veins, carotid arteries, trachea, and esophagus with a sharp knife by a single swipe. This sampling was carried out for organ weight measurement, telomeric DNA determination, telomere regulator gene expression and plasma CORT level at week 4 and week 6 (end of second and fourth weeks of CORT administration). The blood samples due to exsanguination were collected into EDTA tubes and stored in ice. The blood samples were stored at −20 °C prior to DNA extraction. Tissue samples (muscle, liver, and heart) were collected, immediately frozen in liquid nitrogen and then transferred into −80 °C until use. The wet weight of organs (heart, gizzard, adipose tissue, liver, and small intestine) was measured using a sensitive weighing scale as absolute weight during each sampling period per group. Their relative weight was calculated as percentage organ weight per body weight accordingly. Tissues from muscle, liver and heart were collected and immediately stored in liquid nitrogen and then transferred into −80 °C freezer. 

### 2.4. Plasma Corticosterone Level Determination

To ensure undisturbed CORT plasma level, randomly selected chickens were carefully captured, weighed, and slaughtered within 3 min before weighing the rest of the chickens. Five (5) mL blood samples were placed into EDTA tubes. The plasma and erythrocyte were separated by centrifuging at 3000 rpm for 30 min at 25 °C and then stored at −80 °C until they were used for hormone assay. Blank, standard and test sample wells were set and run in duplicate, respectively. The enzyme-linked immunosorbent assay (ELISA) protocol described by the manufacturer (Qayee Biotechnology Co. Ltd., Shanghai, China) was employed for this assay. The final measurement was determined by using a spectrophotometer (Multiskan Go, Thermo Scientific, Waltham, MA, USA). Standard concentration and corresponding OD values were used to calculate the samples’ corticosterone concentrations.

### 2.5. Determination of Telomere Length Using Real-Time Quantitative PCR (qRT-PCR) Analysis

DNA was extracted from the whole blood and tissue samples (muscle, liver and heart) using the blood and tissue DNA innuPREP Mini Kit (Analytik Jena, Jena, Germany) following the recommendations of the manufacturer. The qualities of the DNA extracts were tested using gel electrophoresis and nanodrop (Multiskan Go by Thermo Scientific, USA). Samples with 1.8–2.0 (260/280 ratio) values were stored in a −20 °C freezer prior to the telomeric length determination analysis. For the discovery of the telomere length, the primers were adopted from an available report [36] (Table 2). The housekeeping gene, glyceraldehyde-3-phosphatase (*GAPDH*) [37] primer sequences were designed and sequenced using information contained in Genbank (NCBI) specific to chicken (Table 2). The primers were tested with DNA amplified using MyTaq Red Mix (Bioline, London, UK) with the aid of the polymerase chain reaction (PTC-100TM, Marshall Scientific, New Hampshire, USA) before being run for the qPCR analysis. Twenty nanograms of DNA template was used for both the telomere and the *GAPDH* reactions. The forward and reverse primer concentrations for both telomere and *GAPDH* were 2 µM of each. The primers were mixed with 10 µL SensiFAST SYBR No-ROX qPCR master mix (Bioline, London, UK) for total volume of 20 µL. Ten-fold serial dilutions were performed to obtain standard curves for both the telomere and the housekeeping gene. The samples were arranged accordingly in the PCR machine with identifiers including the non-template control (NTC). The cycling conditions for both telomere and the single copy gene (SCG), *GAPDH* were: 10 min at 95 °C, followed by 40 cycles of 95 °C for 15 s, 60 °C for 1 min, followed by a dissociation (or melt) curve using CFX96 Real-Time PCR System (Bio-Rad, Hercules, CA, USA). Any cycle threshold (Ct) value of standard deviation above one was not used for these analyses. The amplification results (Ct values) were subjected to a Microsoft Excel program designed from standard curves generated to obtain copy numbers in kilobase per reaction (Kb/reaction) of both the telomere and the SCG. The kb/reaction values were then used to calculate the total telomere length in kb per chicken diploid genome according to available information [38].

### 2.6. Gene Expression Analysis of Telomere Length Regulatory Genes

RNA samples were extracted from the muscle, liver, heart and hypothalamus according to the manufacturer’s protocols (InnuPREP RNA Mini kit 2.0, Analytik Jena AG, Germany). The qualities of the RNA were tested using Nanodrop (Multiskan Go by Thermo Scientific, USA) and RNA with 1.8–2.0 (260/280) values was stored at −80 °C for further analysis. The cDNA was synthesized from the extracted RNA (1 µg) samples using the SensiFast cDNA synthesis kit according to the manufacturer’s protocol (Bioline USA Inc., Memphis, TN, USA) with a PCR machine (PTC-100TM, MJ Research Inc., Quebec, Canada) and stored at −20 °C. The cycling conditions for the reverse transcription were: 10 min at 25 °C for annealing, followed by 15 min at 45 °C of reverse transcription, 5 min at 85 °C of inactivation and infinity at 4 °C. The gene expression analysis of chicken telomerase reverse transcriptase (*chTERT*), telomere maintenance gene (*TELO2*), telomeric repeat-containing RNA (*TERRA*), heat shock transcriptional factor 1 (*HSF1*), telomeric repeat transcriptional factor 1 (*TRF1*) and *GAPDH* as the housekeeping gene [37] was obtained by qRT-PCR using the cDNA synthesized from the RNA extracted from the liver, muscle and heart tissues. The primers of these genes specific to chicken were designed and sequenced using information contained in Genbank (NCBI) (Table 2 above). Both the target and the housekeeping genes were simultaneously run in a 96-well plate in duplicates (BIORAD CFX-96, Bio-Rad, CA, USA). The concentrations of the primers for the target and the housekeeping genes were determined by titration; 2 forward and 2 reverse primers were used. The SensiFast Sybr No-ROX kit (Bioline, USA) was used for the amplification of the cDNA. The cycling conditions for the target genes and housekeeping gene were: 10 min at 95 °C, followed by 40 cycles of 95 °C for 15 s, 60 °C for 1 min, followed by a dissociation (or melt) curve using CFX96 Real-Time PCR System (BIO-RAD, USA). Any Ct value of standard deviation above one was not used for further analyses. The mean fold change in the expressions of the target genes at each time was calculated with 2^−ΔΔCT^ where ΔΔCt = (Ct, Target, test – Ct, reference, test) – (Ct, target, Control – Ct, reference, control) [39].

### 2.7. Statistical Analysis

Data on body performance, feed consumption, weight gain and FCR were analyses using repeated measure of ANOVA with SAS 9.4 software [40]. Absolute and relative weight of organs and telomere length data were analyzed using general linear model of SAS 9.4 and 2 × 2 factorial analysis. Means were separated using the Duncan Multiple Range test. Comparison between ages and telomere regulatory genes of the tested chickens and the control was subjected to the *t*-test procedure of the general linear model using SAS 9.4 software. Mortality was analyzed using chi-square test of SAS. All statistical tests were conducted at 95% confidence level.

## 3. Result

### 3.1. Growth Performance and Mortality

Throughout the CORT administration period, CORT treatment significantly (*p* < 0.05) suppressed body weight, feed consumption and weight gain (Table 3). During the trial phases, CORT treatment led to significantly (*p* < 0.05) higher FCR and higher mortality rate in the CORT-fed chicken than the control. No significant difference was observed in body weight, feed consumption, weight gained and FCR between the two groups before the commencement of CORT administration (week 2).

### 3.2. Absolute Weight of Organs

Significant (*p* < 0.05) interaction between age and CORT treatment was noted for absolute weight of the small intestine but not for the heart, liver, abdominal fat, and gizzard (Table 4). After week 4, there was significant (*p* < 0.05) reduction in absolute weight for the small intestine in CORT-fed chicken compared to the control. Significant (*p* < 0.05) effect of age was noted on both control and the CORT-fed chicken. Effect of treatment was significant (*p* < 0.05) on abdominal fat and gizzard sizes but not on heart size. Age significantly (*p* < 0.05) affected heart, liver and abdominal fat sizes but did not affect gizzard size.

### 3.3. Relative Weight of Organs

Significant (*p* < 0.05) effect of treatment was noted for heart, liver, small intestine, abdominal fat, and gizzard relative weights (Table 5). CORT significantly (*p* < 0.05) led to an increase in relative weights of organs at both weeks 4 and 6. No significant interaction was noted between the treatment and age for all the organs’ relative weight. Effect of age was noted on relative weight of liver, abdominal fat and gizzard but was not noted on heart and small intestine relative weights.

### 3.4. Plasma Level of Corticosterone

No significant interaction between age and the CORT treatment was noted for the plasma CORT level in this study (Table 6). However, CORT administration significantly (*p* < 0.05) elevated circulating plasma CORT level in the CORT-fed chicken. No effect of age was noted for plasma CORT level in both the control and the CORT-fed chicken.

### 3.5. Absolute Telomere Length

Significant (*p* < 0.05) interaction effect between CORT treatment and age was noted on telomere length for liver and heart but not for whole blood and muscle (Table 7). CORT treatment significantly (*p* < 0.05) shortened liver and heart telomere length at week 4 but not at week 6 of age. As the CORT feeding advanced, telomere length for liver and the heart increased significantly (*p* < 0.05) in the CORT-fed chicken, making the telomere length of the two groups statistically the same at week 6. Telomere length for muscle was significantly (*p* < 0.05) affected by treatment but was not affected by age. Both treatment and age significantly (*p* < 0.05) affected whole blood telomere length. In addition, telomere length in whole blood decreased, both in the CORT-treated and untreated chicken from week 4 to 6.

### 3.6. Telomere Regulatory Genes

#### 3.6.1. Telomeric Repeat Transcriptional Factor 1 (*TRF1*)

**Figure 1:**
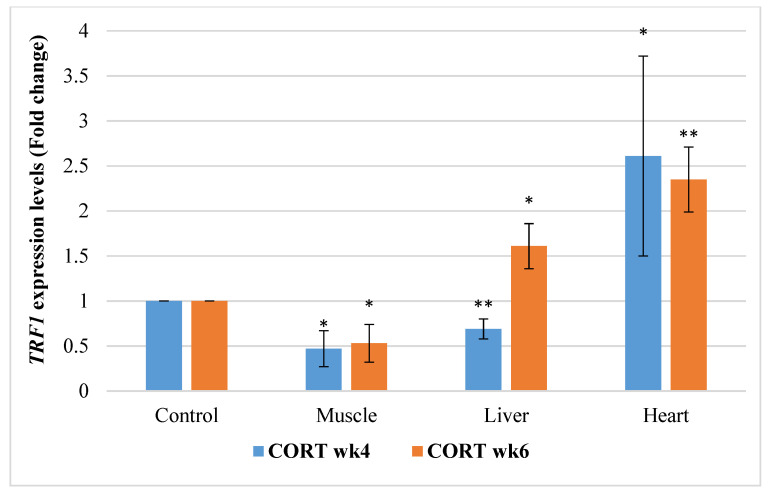
Expression profile of telomeric repeat transcription factor 1 (*TRF1*) in the muscle, liver, and heart at week 4 and 6 of corticosterone-fed chickens compared to the control (in fold change). CORT wk4 = 4 weeks of age (2 weeks of CORT administration); CORT wk6 = 6 weeks of age (4 weeks of CORT administration); n = 20. Probability, * = *p* < 0.05; ** = *p* < 0.01.

The gene expressions of *TRF1* in this study revealed that effects of CORT and duration were significant (Figure 1). It was observed that *TRF1* was significantly downregulated (*p* < 0.05) in the muscle at both week 4 and 6 and in the liver at week 6. However, it was significantly upregulated in the heart at week 4 of CORT treatment. *TRF1* was significantly upregulated in the liver and heart at week 6.

#### 3.6.2. Chicken Telomerase Reverse Transcription Factors (*chTERT*)

**Figure 2:**
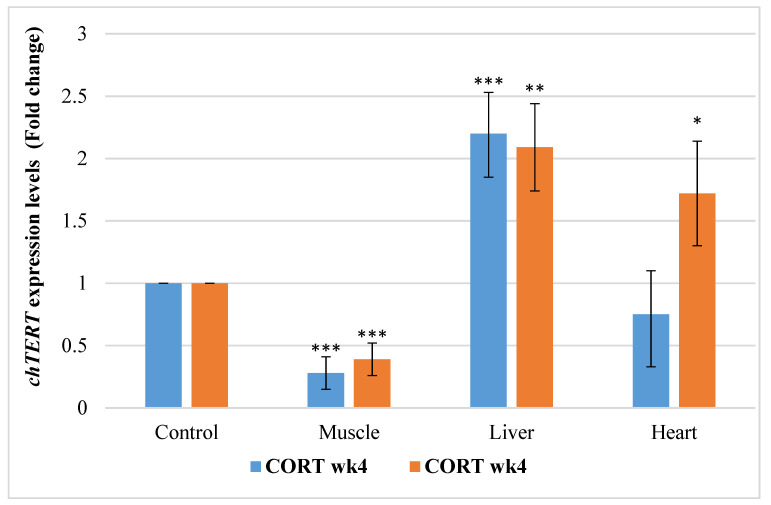
Expression profile of chicken telomerase (*chTERT*) in the muscle, liver, and heart at week 4 and 6 of age in CORT-fed chickens compared to the control. CORT wk4 = 4 weeks of age (2 weeks of CORT administration); CORT wk6 = 6 weeks of age (4 weeks of CORT administration); n = 20. Probability, * = *p* < 0.05; ** = *p* < 0.01; *** = *p* < 0.001.

The gene expression profile of chicken telomerase (*chTERT*) in this study revealed that CORT and duration had a significant effect (Figure 2). *chTERT* was downregulated in the muscle at week 4 and 6 of age (2 and 4 weeks of CORT administration). However, *chTERT* was upregulated at week 4 and 6 in the liver, and at week 6 in the heart of CORT-fed chickens. It was downregulated in the heart at week 4 of the treatment.

#### 3.6.3. Telomere Maintenance Gene 2 (*TELO2*)

**Figure 3:**
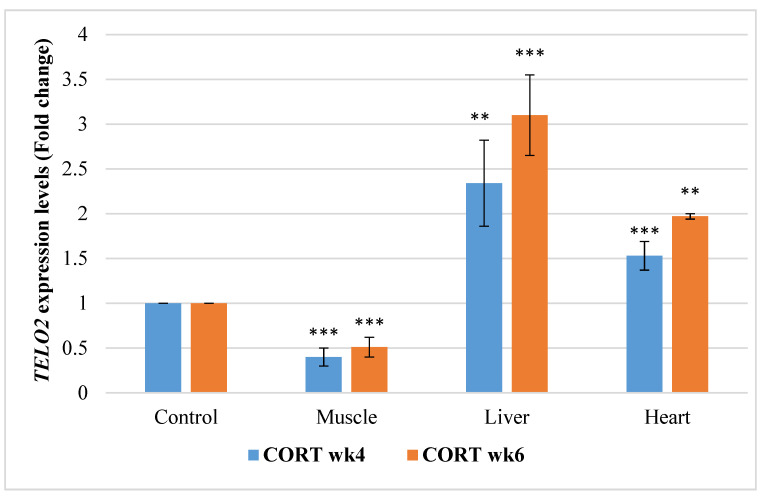
Expression profile of telomere maintenance gene 2 (*TELO2*) in the muscle, liver, and heart at week 4 and 6 of corticosterone-fed chickens compared to the control (in fold change). CORT wk4 = 4 weeks of age (2 weeks of CORT administration); CORT wk6 = 6 weeks of age (4 weeks of CORT administration); n = 20. Probability, * = *p* < 0.05; ** = *p* < 0.01; *** = *p* < 0.001.

The gene expressions of *TELO2* are presented in Figure 3. *TELO2* was significantly downregulated in the muscle of the CORT-fed chickens at weeks 4 and 6. However, *TELO2* was significantly upregulated at weeks 4 and 6 in the liver and heart of the CORT-fed chickens.

#### 3.6.4. Telomeric Repeat-Containing RNA (*TERRA*)

**Figure 4:**
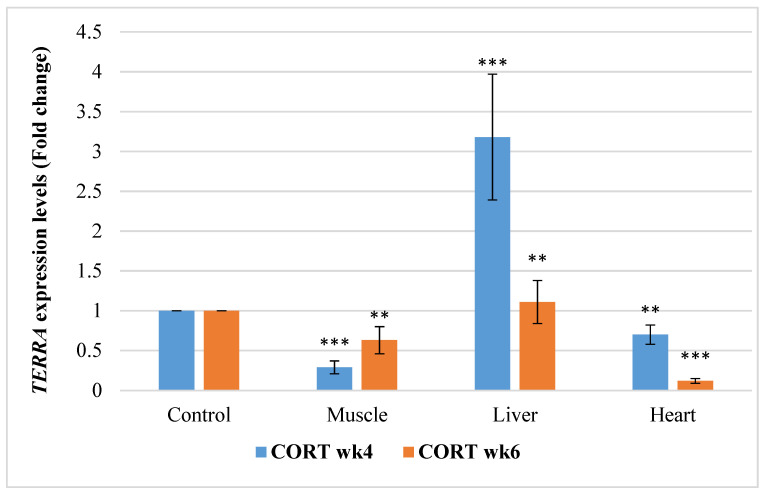
Expression profile of telomeric repeat-containing RNA (*TERRA*) in the muscle, liver, and heart at week 4 and 6 of corticosterone-fed chickens compared to the control (fold change). CORT wk4 = 4 weeks of age (2 weeks of CORT administration); CORT wk6 = 6 weeks of age (4 weeks of CORT administration); n = 20. Probability, ** = *p* < 0.01; *** = *p* < 0.001.

The gene expressions of *TERRA* in this study revealed that impacts of CORT and duration were significant (Figure 4). *TERRA* was downregulated significantly (*p* < 0.05) in the muscle and heart tissue of the CORT-fed chickens at weeks 4 and 6 of CORT administration. Despite this, *TERRA* was upregulated in the liver at week 4 and 6.

#### 3.6.5. Heat Shock Transcriptional Factor 1 (*HSF1*)

**Figure 5:**
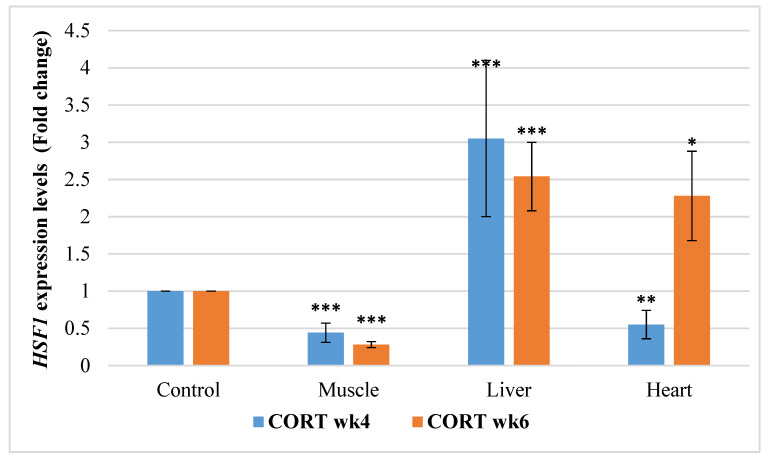
Expression profile of heat shock transcriptional factor 1 (*HSF1*) in the muscle, liver, and heart at week 4 and 6 of corticosterone-fed chickens compared to the control (fold change). CORT wk4 = 4 weeks of age (2 weeks of CORT administration); CORT wk6 = 6 weeks of age (4 weeks of CORT administration); n = 20. Probability, * = *p* < 0.05; ** = *p* < 0.01; *** = *p* < 0.001.

The gene expressions of *HSF1* in this study revealed that impacts of CORT and duration were significant (Figure 5). It was significantly downregulated in the muscle of the CORT-fed chickens at weeks 4 and 6 but was upregulated in the liver of the CORT-fed chickens at weeks 4 and 6. It was, however, downregulated in the heart at week 4 but upregulated at week 6 of the CORT duration.

## 4. Discussion

### 4.1. Growth Performance

Alterations in the performances of animals due to stress could affect telomere lengths. The results from this study indicated that the administration of corticosterone suppressed body weight and body weight gain, and this agreed with some literature findings [35,41,42]. The weight loss in the CORT-fed chicken could be attributed to activated gluconeogenesis and protein breakdown [43,44] and lower feed consumption. FCR was reduced by CORT administration in the CORT-fed chicken and this could be as a result of poor feed assimilation in the body. The findings revealed that the CORT-fed chicken diverted nutrients meant for growth performance to lipid accumulation and fatty livers [45]. In addition, the poor growth and high mortality rate in the CORT-fed chicken could be attributed to the accumulation of reactive oxygen species (ROS) [18]. The higher mortality in the CORT-fed chicken is expected and this emphasized the association between chronic stress and the increase in mortality rate in broiler chicken.

### 4.2. Absolute and Relative Weights of Organ

The health status of animals can be diagnosed using their organs [35]. In the present study, we observed a very interesting interaction effect between CORT treatment and age on small intestinal absolute weight. This interaction was evident when CORT-fed chicken exhibited substantial reduction in small intestinal weight at week 6 compared to the control group. In contrast, CORT did not significantly influence small intestinal weight at week 4. CORT had been reported to reduce wet weight of the small intestine in a finding [35]. The small intestine aids in growth and animal performance [46]. The reduction in the absolute weight of the small intestine could lead to a low proportion of nutrient uptake and corresponding loss of protein [35]. According to the present results, there was significant suppression of the absolute weight of the liver, abdominal fat and gizzard due to CORT treatment. Significant improvement in the weights of heart, liver, small intestine, and abdominal fat was noted with advancement in age. Reduction in the sizes of these organs could be attributed to insufficient nutrient uptake in the CORT-treated chicken. Our study revealed that CORT significantly increased the relative weights of heart, small intestine, abdominal fat and gizzard. Relative weights of liver and gizzard significantly reduced with advancement in age but increased in abdominal fat. Increase in the relative weights of the small intestine, liver, and liver fat due to CORT administration in chicken had been reported in most studies [35,41]. This improvement in the relative sizes of organs could be attributed to inflammation acquired during CORT administration. The relationship between the organ’s weight and the telomere length has not been uncovered. However, these organs are described as metabolically active with a positive correlation with body weights [20]. Increased metabolic activities could amount to high ROS which is noted to trigger chronic oxidative damage [6] and hence telomere length attrition [19].

### 4.3. Plasma Corticosterone Levels

Administration of CORT through feeding or implantation had been reported to increase the plasma CORT levels [42]. Meanwhile, influence of CORT treatment and administration period (short versus long) on glucocorticoids had been reported [1]. In the current study, we revealed that CORT administration significantly elevated plasma CORT level and this change was independent of age. Similar levels of CORT plasma had been noticed between CORT-fed sparrows and the control after a week, but the CORT level returned to baseline level two months after implantation [47]. The researchers [47], however, revealed that CORT implantation induced a short-lived negative effect on body weight, blood, and feather weight. In contrast, our data in the current study revealed that influence of CORT administration persisted on the plasma CORT levels, growth performance and other physiological components. In view of removing controversies surrounding the use of CORT as a biomarker of animal welfare due to its inconsistency, telomere length as a more reliable and conserved biomarker of stress is proposed as an alternative in this study.

### 4.4. Telomere Length

Telomere is a nucleoprotein protecting the end of the chromosome from degradation during cell division and it is highly sensitive to oxidative attack due to its high guanine content [48], the characteristic that prolongs its recovery and gives it stable potential to measure stress conditions. Telomere length is usually shortened with stress and advancement in age and could be used as a biomarker of stress. Its shortening rate predicts a species life span [49]. In the present study, we noticed a noteworthy interaction between the CORT treatment and age for liver and heart telomere length. These interactions were evident when the CORT-fed chicken revealed a drastic loss in liver and heart telomere length at week 4. On the contrary, CORT did not influence liver and heart telomere length at week 6. These results suggest that liver and heart telomere length are more susceptible to CORT treatment at week 4 than week 6. The telomeric DNA shortening in the heart and liver at week 4 was a result of interaction between CORT treatment and age. The telomere length attrition at this early stage of induction could be attributed to the REE and high metabolic rates of these organs [20,21] combined with chronic oxidative damage due to CORT administration [18]. In addition, with advancement in age, liver and heart telomere length improved in the CORT-fed chicken. Based on the current findings, CORT treatment significantly caused telomeric attrition in whole blood and muscle. Influence of age of induction was noticed on whole blood telomere length but was not observed in the muscle. Oxidative stress induced by CORT could be responsible for the poor performance obtained in the CORT-fed chickens and this could be attributed to damaged proteins and DNA [50]. In this study, chickens with short telomere lengths revealed suppressed body weights. The production of ROS via CORT administration usually affects the dynamics of the telomeres [18]. Telomere length has been used to measure individual fitness and survivability [51]. Moreover, the effect of age in 178 single-comb White Leghorns from 10 weeks old was reported for lymphocytes [52]. The amount of telomeric DNA was observed to decrease with the advancement in age in this study. When cell ageing sets in, inadequate amount or absence of some restorative genes will be experienced, leading to aggravated telomeric shortening at senescence [26,53].

Telomere length could be implicated in the high mortality observed in CORT-fed chickens. Reports have attributed telomere length to adaptation and survivability [54,55]. High risk of mortality and low life expectancy have been related to short telomere length [56,57]. Telomere length was sometimes related to longevity and reproductive performances in various studies [13]. In the current study, variation of the telomere in liver, heart and muscle were not affected by age. This implies that telomere length of the tissues could be restored due to the activities of telomerase and the shelterin genes which are usually activated in tumor cells [24,58,59]. This implies that increased telomere length could sometimes be an indicator of unhealthy conditions like cancers. The loss in telomere length in the whole blood due to age could be a result of diminished levels of telomerase which are usually low in somatic tissues [19,60]. Our results implied that with increased duration of treatment, CORT administration could lead to telomere length improvement in tissues suffering from tumors such as liver and heart [24,61]. This improvement in telomere length could be attributed to telomerase activities which are usually higher in damaged or cancer cells and hence maintain telomere lengths [28].

Surprisingly, there was a decline in the telomere length for blood, liver, and the heart from week 4 to week 6 of age in the untreated chickens. Muscle did not reveal such a difference. The difference in the characteristics of telomere length could be attributed to a genetic and tissue effect. Telomere length was previously associated with genetic and non-genetic factors [61]. The results in this study imply that chicken blood, liver and heart telomere length are prone to shortening and this could be the reason for major liver and heart-related diseases usually reported in chicken. Modern strains of broiler chicken have been reported to be highly susceptible to heart failure and heart-related mortalities such as ascites and sudden death syndrome [62]. Short telomere length, to make it clear, has been implicated as the main cause of heart disease and other age-associated diseases [63]. In fact, liver fibrosis and cirrhosis have been reported to be caused by critically short telomeres, a phenomenon known as DNA damage response [64]. Decline in the telomere length has been reported in the human heart along the gestation period and heart development while in other tissue, such as kidney and liver, telomere length remains unchanged [65]. We therefore discovered in this study that age, when combined with stress, could lead to drastic telomere length attrition. Telomere length has been reported to reduce with age in fishes, reptiles, and humans, except in water python and Leach’s petrel [66]. Telomere length could therefore be considered as an indicator of aging. Relationships between telomere length and stress [67] hint that telomere length might not be used only for an indicator of chronological age [68], but also for a marker of organism lifestyle [69] and a good proxy of organism fitness or biological age [70]. Here, we reported higher abdominal fat in CORT-fed chicken and increased fat in the untreated chicken with an increase in age. Increase in fat deposit could lead to obesity, ROS, oxidative damage and non-alcoholic fatty liver and these are risk factors for short telomere length.

### 4.5. Telomere Length Regulatory Genes

The results of the telomere regulatory genes revealed that they all influenced telomere length integrity. They were all implicated in the telomere length shortenings in the blood and the tissues. The expressions of these genes are tissue-dependent. The high variabilities observed in the liver and heart samples showed that these genes vary from cell to cell. It happens in the group of stem cells and cancer or tumor cells. These high variabilities are the cumulative results of intrinsic genetic (inherent) factors, extrinsic factors, and stochastic factors. It has been reported that cell-to-cell variations are often observed within cancerous and embryonic cell samples [71].

In muscle, the shortening of telomere length could be a result of the downregulation of *TRF1, chTERT, TELO2, TERRA* and *HSF1* due to CORT administration. A study has revealed that *TERRA* was involved in telomerase nucleation [72]. The results indicated that the expressions of the telomere regulators were affected by CORT and age. The *TRF1* is one of the components of the shelterin complex that shelters the telomere. Telomerase activities are dependent on the nucleotide sequences from this complex for telomeric DNA formation [72]. Findings suggested that *TRF1* mediated telomere length and function [65]. The downregulation of the *TRF1* by chronic stress in the CORT-fed chickens could affect the telomeric DNA. This effect might initiate chromosomal instability [73]. The deficiency of *TRF1, TELO2, TERRA* and *HSF1* in the muscle could be responsible for the deficiency in the *chTERT* and hence the telomere length shortening due to their reduced nucleation to the *chTERT* [32,59,74]. In the current study, TRF1 was downregulated in muscle. It has been reported that *TRF1* was low in mice skeletal muscle exposed to treadmill running bout stress. The reduced *TRF1* brought about a significant increase in the apoptosis mediator, *P38 MAPK* phosphorylation [75]. The upregulation of *TRF1* led to stability in the liver telomere at week 6, as this gene specializes in promoting replication of telomeres [76]. This implied that in the liver and heart, *TRF1* could be a negative regulator and this assertion agreed with the report obtained in study [73]. Telomerase was not increased by *TRF1* in the heart at week 4, probably as a result of downregulation of *TERRA* and *HSF1.* It has been revealed that when *TRF1* was genetically or chemically inhibited in mouse, increased telomeric DNA damage, reduced produced proliferation and stemness were reported [76,77]. The telomere maintenance gene 2 (*TELO2*) might have a part in telomere length regulation and maintenance as it was found in the pathway of telomerase and in the complex responsible for cellular resistance against DNA damage stress, especially due to radiation, ultraviolet and mitomycin [30,78]. It is being examined and associated with organs and other telomere-regulating genes and we discovered that it has good links with these genes and could be a good candidate for telomeric studies. Moreover, *TERRA* is another gene that plays a pivotal role in the mediation of the heterochromatic marks in the remodeling complex [79]. *TERRA* has been reported to be involved in telomerase nucleation [72]. Though its expression in tissues has not been detailed, its function in telomeric DNA upon heat stress in fibroblast has been revealed [32]. Studies revealed that telomerase activity was very low in almost all somatic tissues or cells with high proliferative potential [59,74]. Cells deficient in *HSF1* had been reported to be deficient in *TERRA* and hence had short telomere lengths [32]. Koskas et al. [32] further reported that *HSF1* promoted *TERRA* transcription and telomere length protection upon heat stress. *HSF1* has been classed among the cancer-related genes group. It is mostly expressed in all tissues in the human body. When the telomere maintenance mechanism was deficient, increased telomere transcription was reported to result in telomere shortening due to DNA replication–dependent loss of the telomere pathways [80].

Telomerase (*TERT*) function is usually high in gamete, stem, and tumor cells. It has been reported to be low in adult somatic cells in mice, whereas it is completely absent in human adult somatic cells [81]. Higher activity of *TERT* has been reported in cancer cells and in most cells suffering from tumors [82] while *TRF1* provided a nucleotide sequence for *TERT*. The heart is made up of cardiac stem cells which help in the self-regeneration of the heart due to high telomerase activities [83]. In addition, activation of *TELO2, HSF1* and *TERRA* in the liver could be responsible for the maintenance of the telomere length in the liver at week 6 in the current study. Telomere shortening was noted to induce *TERRA* expression which then activated telomerase nucleation [72] which could then initiate telomere recovery. Moreover, *HSF1* has been reported to promote *TERRA* transcription and telomere length protection upon heat stress [32]. The observation in this study suggests that *TELO2* could therefore be part of the telomerase pathway, which implies that *TELO2* is a typical novel gene which can be used as a stress biomarker and hence can be considered as a mechanism for telomere synthesis. This observation agreed with the information available in the rat genome database which revealed that *TELO2* was shown to maintain the telomere via telomerase [78] and its role in the telomeric-binding activities had been suggested [30]. The upregulation of *TERRA* in the liver was expected as it was mobilized to areas with short telomeres to cause the nucleation of telomerase. Interactions of *TERRA* with TRFs could mobilize *TERRA* to the telomeres, causing *TERRA* R-loops to be synthesized at the severely shortened telomere, thereby preventing the DNA damage response [31,84]. This observation was noticed in the liver but not in the muscle in the current study. The differences observed in the tissues could be attributed to tissue peculiarities which implied that they have diverse characteristics. Furthermore, *HSF1* activated *TERRA* in the liver but not in the heart. Higher levels of reactive oxygen species (ROS), cleaved caspases and fragmented DNA in the gastrocnemius were reported in muscles of heat-exposed mice but not in control mice and these changes were not observed in the livers of heat-exposed mice [85]. The reason for the downregulation of *TERRA* in the heart and the muscle could be as a result of mutations, as earlier elucidated [86,87].

Generally, telomere length is progressively lost in muscle due to inability of DNA polymerase to completely replicate telomere under stress [26]. The loss in telomere could also be attributed to absence or lower telomere restoration factors such as telomerase activities, shelterin protein complex [24,58], *TERRA* and *HSF1* [32] that regulate the telomeres. These restoration factors tend to be activated in the liver and heart.

## 5. Conclusions

It was observed that CORT elevated plasma CORT level and altered performances and organ sizes in the CORT-treated chicken, suggesting that both poor conditions were caused by chronic oxidative stress. CORT led to telomere length attrition and altered the tissue telomere length regulators in the CORT-fed group. The expressions of *TRF1, chTERT, TELO2, TERRA* and *HSF1* affected telomere length behavior under chronic stress. The gene expression reports revealed that muscle tissue could be more susceptible to chronic stress and telomeric DNA attritions. Telomere loss in blood is age-dependent, suggesting that it is a potential biomarker of aging. In conclusion, telomere length and its regulators are diverse and can be used as novel biomarkers of stress in broiler chickens.

## Figures and Tables

**Table 1:** Nutrient compositions of commercial broiler starter and finisher diets.

Composition	Starter ^1^	Finisher ^2^
Crude protein (%)	23.00	19.00
Crude fiber (%)	5.00	5.00
Crude fat (%)	5.00	5.00
Moisture (%)	13.00	13.00
Ash (%)	8.00	8.00
Calcium (%)	0.80	0.80
Phosphorous (%)	0.40	0.40

^1^ Commercial starter diet for broiler in crumble form. ^2^ Commercial finisher diet for broiler in pellet form.

**Table 2:** Primer sequences for telomere and telomere regulatory genes in chicken.

No	Gene	Primers’ Sequence	Accession Numbers	Amplicon Sizes (bp)
1	Telomere	F—GGTTTTTGAGGGTGAGGGTGAGGGTGAGGGTGAGGGT R—TCCCGACTATCCCTATCCCTATCCCTATCCCTATCCCTA	NA	79
2	*TRF1*	F—GGAGGAACGGTTTCCCTAAG R—CTGATGCTGCCCACAGTAGA	NC_-_006089.5	178
3	*TERRA*	F—GGCCACTGTAAATGGCTGTT R—GTTTGCACAAGGGTCTCCAT	NC_-_006127.5	219
4	*HSF1*	F—TCTCTGGGTGTCCTTCTGCT R—CTCCTTCCACAGAGCACCTC	NC_-_006089.5	151
5	*TELO2*	F—GGATGACCCTCAGAGATGGA R—ATTGGTGTGACCAGGAAAGCe	NC_-_006101.5	249
6	*chTERT*	F—AGGTGCCCAAAACTGAACAC R—CTTCCAAGGGAGACTTGCAG	NC_-_006089.5	184
7	*GAPDH*	F—ACTATGCGGTTCCCAGTGTC R—TGCCACCATCAGAAAAATGA	NC_-_006088.5	215

NA = not available.

**Table 3:** Effects of corticosterone administration on body weight, feed consumption and feed conversion ratio (FCR) of broiler chicken.

Age/Traits	CTRL	CORT-Fed Chicken	SEM	*p*-Values
**Body weight (g)**				
Week 2	541.88	539.76	8.76	0.8585
Week 4	1509.00	1054.21	23.48	0.001
Week 6	2363.55	1479.18	34.62	0.001
**Feed consumption** **(g/bird/week)**				
Week 2	422.00	439.80	8.22	0.1730
Week 4	990.00	843.30	17.65	0.0004
Week 6	1057.70	822.30	22.57	0.0001
**Weight gain (g/bird/week)**				
Week 2	347.56	344.83	5.41	0.735
Week 4	496.42	259.55	17.39	0.0001
Week 6	1056.67	822.33	22.57	0.0001
**FCR (feed/gain)**				
Week 2	1.21	1.27	0.02	0.0557
Week 4	2.00	3.27	0.12	0.0001
Week 6	2.27	3.60	0.11	0.0001
**Mortality (Week 0–Week 6)**	1.00	7.00	0.09	0.024

CORT = corticosterone; Week 2 = period of no CORT administration; Week 4 = 2 weeks of CORT administration; Week 6 = 4 weeks of CORT administration. There were 50 observations per treatment.

**Table 4:** Effect of corticosterone feeding and age on absolute organ weight.

Age	Treatment	Heart (g)	Liver (g)	Smallint (g)	AbdFat (g)	Gizzard (g)
**Week 4**	CTRL	7.85 ^b^	36.27 ^c^	76.55 ^c^	17.87 ^d^	29.36 ^b^
	CORT	7.22 ^b^	55.21 ^b^	66.09 ^c^	27.24 ^c^	36.13 ^ab^
**Week 6**	CTRL	11.24 ^a^	49.17 ^b^	123.56 ^a^	52.03 ^b^	29.36 ^b^
	CORT	11.74 ^a^	75.02 ^a^	93.63 ^b^	63.12 ^a^	42.42 ^a^
	SEM	0.54	3.42	2.48	2.48	2.95
***p* values**	Age	<0.0001	<0.0001	<0.0001	<0.0001	0.359
	Treatment	0.907	<0.0001	<0.0001	<0.0001	0.004
	Age × Treatment	0.319	0.363	0.041	0.74	0.287

^a,b,c,d^ Means within a column subgroup with no common superscripts are significantly different at *p* < 0.05. SEM = standard error of the mean for main effects (n = 20). Smallint = small intestine; AbdFat = abdominal fat; CTRL = control group; CORT = corticosterone-treated group; Week 4 = 4 weeks of age (2 weeks of CORT administration); Week 6 = 6 weeks of age (4 weeks of CORT administration).

**Table 5:** Effect of corticosterone feeding and age on relative organ weights.

Age	Treatment	Heart (%)	Liver (%)	Smallint (%)	AbdFat (%)	Gizzard (%)
**Week 4**	CTRL	0.50 ^b^	2.31 ^b^	4.88 ^b^	1.14 ^d^	1.90 ^c^
	CORT	0.68 ^a^	5.15 ^a^	6.21 ^a^	2.55 ^b^	3.35 ^a^
**Week 6**	CTRL	0.44 ^b^	1.93 ^b^	4.87 ^b^	2.06 ^c^	1.17 ^d^
	CORT	0.74 ^a^	4.59 ^a^	5.72 ^a^	3.83 ^a^	2.61 ^b^
	SEM	0.03	0.20	0.22	0.14	0.20
***p* values**	Age	0.756	0.041	0.268	<0.0001	0.0016
	Treatment	<0.0001	<0.0001	<0.0001	<0.0001	<0.0001
	Age × Treatment	0.212	0.667	0.282	0.213	0.999

^a,b,c,d^ Means within a column subgroup with no common superscripts are significantly different at *p* < 0.05. SEM = standard error of the mean for main effects (*n* = 20). Smallint = small intestine; AbdFat = abdominal fat; CTRL = control group; CORT = corticosterone-treated group; Week 4 = 4 weeks of age (2 weeks of CORT administration); Week 6 = 6 weeks of age (4 weeks of CORT administration).

**Table 6:** Effect of corticosterone administration on plasma corticosterone level.

Age	Week 4		Week 6			*p* Values		
Treatment	CTRL	CORT	CTRL	CORT	SEM	Age	Treatment	Age × Treatment
Plasma CORT (ng/mL)	6.80 ^b^	7.65 ^a^	6.73 ^b^	7.94 ^a^	0.37	0.685	0.0004	0.830

^a,b^ Means within a row subgroup with no common superscripts are significantly different at *p* < 0.05. SEM = standard error of the mean for main effects (*n* = 20). CTRL = control group; CORT = corticosterone-treated group; Week 4 = 4 weeks of age (2 weeks of CORT administration); Week 6 = 6 weeks of age (4 weeks of CORT administration).

**Table 7:** Effect of corticosterone feeding and age on absolute telomere length in whole blood, muscle, liver and heart of broiler chicken.

Age	Treatment	Whole Blood	Muscle	Liver	Heart
**Week 4**	CTRL	526.40 ^a^	446.68 ^a^	564.96 ^a^	576.89 ^a^
	CORT	334.50 ^bc^	313.84 ^b^	346.84 ^b^	264.43 ^b^
**Week 6**	CTRL	417.96 ^ab^	463.41 ^a^	481.07 ^ab^	355.72 ^ab^
	CORT	260.09 ^c^	282.39 ^b^	467.59 ^ab^	364.98 ^ab^
	SEM	37.27	54.90	58.33	56.03
***p* values**	Age	0.016	0.779	0.807	0.316
	Treatment	<0.0001	0.005	0.028	0.009
	Age × Treatment	0.921	0.817	0.047	0.018

^a,b^ Means within a column subgroup with no common superscripts are significantly different at *p* < 0.05. SEM = standard error of the mean for main effects (*n* = 20). CTRL = control group; CORT = corticosterone-treated group; Week 4 = 4 weeks of age (2 weeks of CORT administration); Week 6 = 6 weeks of age (4 weeks of CORT administration).

## Data Availability

The data presented in this finding are available upon request from the corresponding author.

## References

[1] Rushen J. (1991). Problems associated with the interpretation of physiological data in the assessment of animal welfare. Appl. Anim. Behav. Sci..

[2] Fairhurst G.D., Marchant T.A., Soos C., Machin K.L., Clark R.G. (2013). Experimental relationships between levels of corticosterone in plasma and feathers in a free-living bird. J. Exp. Biol..

[3] Dhabhar F.S., McEwen B.S. (1997). Acute stress enhances while chronic stress suppresses cell-mediated immunity in vivo: A potential role for leukocyte trafficking. Brain Behav. Immun..

[4] Dhabhar F.S. (2000). Acute stress enhances while chronic stress suppresses skin immunity. The role of stress hormones and leukocyte trafficking. Ann. N. Y. Acad. Sci..

[5] Romero L.M. (2004). Physiological stress in ecology: Lessons from biomedical research. Trends Ecol. Evol..

[6] Spiers J.G., Chen H.C., Sernia C., Lavidis N.A. (2015). Activation of the hypothalamic-pituitary-adrenal stress axis induces cellular oxidative stress. Front. Neurosci..

[7] Bennett M.C., Mlady G.W., Fleshner M., Rose M.G. (1996). Synergy between chronic corticosterone and sodium azide treatments in producing a spatial learning deficit and inhibiting cytochrome oxidase activity. Proc. Natl. Acad. Sci. USA.

[8] Barna J., Csermely P., Vellai T. (2018). Roles of heat shock factor 1 beyond the heat shock response. Cell. Mol. Life Sci..

[9] Jones T.J., Li D., Wolf I.M., Wadekar S.A., Periyasamy S., Sánchez E.R. (2004). Enhancement of glucocorticoid receptor-mediated gene expression by constitutively active heat shock factor 1. Mol. Endocrinol..

[10] Houbon J.M.J., Moonen H.J.J., van Schooter F.J., Hageman G.J. (2007). Telomere length assessment: Biomarkers of chronic oxidative state?. Free Radic. Biol Med..

[11] Moyzis R.K., Buckingham J.M., Cram L.S., Dani M., Deaven L.L., Jones M.D., Meyne J., Ratliff R.L., Wu J.R. (1988). A highly conserved repetitive DNA sequence, (TTAGGG), present at the telomeres of human chromosomes. Proc. Natl. Acad. Sci. USA.

[12] Kotrschal A., Ilmonen P., Penn D.J. (2007). Stress impacts telomere dynamics. Biol. Lett..

[13] Angelier F., Vleck C.M., Holberton R.L., Marra P.P. (2013). Telomere length, non-breeding habitat and return rate in male American redstarts. Funct. Ecol..

[14] Heidinger B.J., Blount J.D., Boner W., Griffiths K., Metcalfe N.B., Monaghan P. (2012). Telomere length in early life predicts lifespan. Proc. Natl. Acad. Sci. USA.

[15] Blackburn E.H., Chan S., Chang J., Fulton T.B., Krauskopf A., Mceachern M., Prescott J., Roy J., Smith C., Wang H. (2000). Molecular manifestations and molecular determinants of telomere capping. Cold Spring Harb. Symp. Quant. Biol..

[16] Richter T., Proctor C. (2007). The role of intracellular peroxide levels on the development and maintenance of telomere-dependent senescence. Exp. Gerontol..

[17] Sohn S.H., Subramani V.K. (2014). Dynamics of telomere length in the chicken. World Poult. Sci. J..

[18] Costantini D., Marasco V., Møller A.P. (2011). A meta-analysis of glucocorticoids as modulators of oxidative stress in vertebrates. J. Comp. Physiol. Biol..

[19] Von Zglinicki T. (2002). Oxidative stress shortens telomeres. Trends Biochem. Sci..

[20] Elia M., Kinney J.M., Tucker H.N. (1992). Organ and tissue contribution to metabolic rate. Energy Metabolism. Tissue Determinants and Cellular Corollaries.

[21] Holliday M.A., Fulkner F., Tanner J.M. (1986). Body composition and energy needs during growth. Human Growth: A Comprehensive Treatise.

[22] Hsu A., Heshka S., Janumala I., Song M., Horlick M., Krasnow N., Gallagher D. (2003). Larger mass of high-metabolic-rate organs does not explain higher resting energy expenditure in children. Am. J. Clin. Nutr..

[23] Bianchi A., Smith S., Chong L., Elias P., de Lange T. (1997). TRF1 is a dimer and bends telomeric DNA. EMBO J..

[24] De Lange T. (2005). Shelterin: The protein complex that shapes and safeguards human telomeres. Genes Dev..

[25] Ye J.Z.S., Donigian J.R., van Overbeek M., Loayza D., Luo Y., Krutchinsky A.N., Chait B.T., de Lange T. (2004). TIN2 binds TRF1 and TRF2 simultaneously and stabilizes the TRF2 complex on telomeres. J. Biol. Chem..

[26] Blackburn E.H. (1991). Structure and function of telomeres. Nature.

[27] Greider C.W., Blackburn E.H. (1985). Identification of a specific telomere terminal transferase activity in Tetrahymena extracts. Cell.

[28] Lansdorp P.M. (2008). Telomeres, stem cells, and hematology. ASH 50th anniversary review. Blood.

[29] Taylor H.A., Delany M.E. (2000). Ontogeny of telomerase in chicken: Impact of downregulation on pre- and postnatal telomere length in vivo. Dev. Growth Diff..

[30] Gaudet P., Livestock M.S., Lweis S.E., Thomas P.D. (2011). Phylogenetic-based propagation of functional annotations, within the Gene ontology consortium. Brief. Bioinform..

[31] Graf M., Bonetti D., Lockhart A., Serhal K., Kellner V., Maicher A., Joilivet P., Teixeira M.T., Luke B. (2017). Telomere length determines TERRA and R-loop regulation through the cell cycle. Cell.

[32] Koskas S., Decottignies A., Dufour S., Pezet M., Verdel A., Vourch C., Faure V. (2017). Heat shock factor 1 promotes TERRA transcription and telomere protection upon heat stress. Nucleic Acids Res..

[33] Blasco M., Schoeftner S. (2008). Developmentally regulated transcription of mammalian telomeres by DNA-dependent RNA polymerase II. Nat. Cell Biol..

[34] Romano G.H., Harari Y., Yehuda T., Podhorzer A., Rubinstein L., Shamir R., Gottlieb A., Silberberg Y., Pe’er D., Ruppin E. (2013). Environmental stresses disrupt telomere length homeostasis. PLoS Genet..

[35] Hu X.F., Guo Y.M., Huang B.Y., Zhang L.B., Bun S., Liu D. (2010). Effect of corticosterone administration on small intestinal weight and expression of small intestinal nutrient transporter mRNA of broiler chickens. Asian Aust. J. Anim. Sci..

[36] Cawthon R.M. (2002). Telomere measurement by quantitative PCR. Nucleic Acids Res..

[37] Herborn K.A., Heidinger B.J., Boner W., Noguera J.C., Adam A., Daunt F., Monaghan P. (2014). Stress exposure in early post-natal life reduces telomere length: An experimental demonstration in a long-lived seabird. Proc. R. Soc. Lond..

[38] O’Callaghan N.J., Fenech M. (2011). A quantitative PCR method for measuring absolute telomere length. BMC.

[39] Livak K.J., Schmittgen T.D. (2001). Analysis of relative gene expression using real-time quantitative PCR and the 2^−ΔΔCT^ method. Methods.

[40] SAS Publishing (2002). Statistical Analysis System Multiple Incorporation. Users Guide Statistical Version.

[41] Gross W.B., Siegel P.B. (1981). Some effects of feeding deoxycorticosterone to chickens. Poult. Sci..

[42] Zulkifli I., Najafi P., Nurfarahin A.J., Soleimani A.F., Kumari S., Anna Aryan A., O’ Reilly E.L., Eckersalli P.D. (2014). Acute phase proteins, interleukin 6, and heat shock protein 70 in broiler chickens administered with corticosterone. Poult. Sci..

[43] Lin H., Sui J., Jiao H., Buyse H. (2006). Impaired development of broiler chickens by stress mimicked by corticosterone exposure. Comp. Biochem. Phys. A.

[44] Hayashi K., Kaneda S., Otsuka A., Tomita Y. Effects of ambient temperature and thyroxine on protein turnover and oxygen consumption inchicken squeletal muscle. Proceedings of the 19th World’s Poultry Congress.

[45] Gross W.B., Siegel P.B., DuBose R.T. (1980). Some effects of feeding corticosterone to chicken. Poult. Sci..

[46] Ziegler T.R., Evans M.E., Fernandez-Estivariz C., Jones D.P. (2003). Trophic and cytoprotective nutrition for intestinal adaptation, mucosal repair, and barrier function. Annu. Rev. Nutr..

[47] Vágási C.I., Pătraș L., Pap P.L., Vincze O., Mureșan C., Németh J., Lendvai Á.Z. (2018). Experimental increase in baseline corticosterone level reduces oxidative damage and enhances innate immune response. PONE.

[48] Kawanishi S., Oikawa S. (2004). Mechanism of telomere shortening by oxidative stress. Ann. N. Y. Acad. Sci..

[49] Kurt W., Vera E., Martinez-nevado E., Sanpera C., Blasco M.A. (2019). Telomere shortening rate predicts species life span. Proc. Natl. Acad. Sci. USA.

[50] Klaunig J.E., Zemin W., Xinzhu P., Shaoyu Z. (2011). Oxidative stress and oxidative damage in chemical carcinogenesis and multistage carcinogenesis. Toxicol. Appl. Pharmacol..

[51] Reichert S. (2013). Determinants of Telomere Length and Implications in Life History Trade-Offs. Ph.D. Thesis.

[52] Kim Y.J., Subramani V.K., Sohn S.H. (2011). Age prediction in the chickens using telomere quantity by quantitative fluorescence in situ hybridisation technique. Asian Aust. J. Anim. Sci..

[53] Harley C.B., Futcher A.B., Greider C.W. (1990). Telomeres shorten during ageing of human fibroblasts. Nature.

[54] Martin T.E., Riordan M.M., Repin R., Mouton J.C., Blake W.M., Fleishman E. (2017). Apparent annual survival estimates of tropical songbirds better reflect life history variation when based on intensive field methods. Glob. Ecol. Biogeogr..

[55] Muñoz A.P., Kéry M., Martins P.V., Ferraz G. (2018). Age effects on survival of Amazon forest birds and the latitudinal gradient in bird survival. Auk Ornithol. Adv..

[56] Wilbourn R.V., Moatt J.P., Froy H., Wally C.A., Nussey D.H., Broonekamp J.J. (2018). The relationship between telomere length and mortality Risk in non-model vertebrate system: A Meta Analysis. Phil. Trans. R. Soc. B Biol. Sci..

[57] Tricola G.M., Simons M.J.P., Atema E., Boughton R.K., Brown J.L., Dearborn D.C., Divoky G., Eimes J.A., Huntington C.E., Kitaysky A.E. (2018). The rate of telomere loss is related to maximum lifespan in birds. Phil. Trans. R. Soc. B Biol. Sci..

[58] Blackburn E.H. (2001). Switching and signalling at the telomere. Cell.

[59] Dunham M.A., Neumann A.A., Fasching C.L., Reddel R.R. (2000). Telomere maintenance by recombination in human cells. Nat. Genet..

[60] Greider C.W. (1998). Telomerase activity, cell proliferation, and cancer. Proc. Natl. Acad. Sci. USA.

[61] Martinez P., Blasco M.A. (2018). Heart-Breaking telomeres. Circ. Res..

[62] Olkowski A.A. (2007). Pathophysiology of heart failure in broiler chickens: Structural, biochemical and molecular characteristics. Poult. Sci..

[63] Strong M.A., Vidal-Cadenas S.L., Karim B., Yu H., Guo N., Greider C.W. (2011). Phenotypes in mTERT^+^/^−^ and mTERT^-^/^-^ mice are due to short telomeres, not telomere-independent functions of telomerase reverse transcriptase. Mol. Cell Biol..

[64] Armanios M. (2013). Telomeres and age-related disease: How telomere biology informs clinical paradigm. J. Clin. Investig..

[65] Booth S.A., Charchar F.J. (2017). Cardiac telomere length in heart development, function and disease. Physiol. Genom..

[66] Yip B.W., Mok H.O., Peterson D.R., Wan M.T., Taniguchi Y., Ge W., Au D.W. (2017). Sex-dependent telomere shortening, telomerase activity and oxidative damage in marine medaka Oryzias melastigma during aging. Mar. Pollut. Bull..

[67] Epel E.S., Blackburn E.H., Lin J., Dhabhar F.S., Adler N.E., Morrow J.D., Cawthon R.M. (2004). Accelerated telomere shortening in response to life stress. Proc. Natl. Acad. Sci. USA.

[68] Monaghan P., Haussmann M.F. (2006). Do telomere dynamics link lifestyle and lifespan?. Trends Ecol. Evol..

[69] Monaghan P. (2010). Telomeres and life histories: The long and the short of it. Ann. N. Y. Acad. Sci..

[70] Bauch C., Becker P.H., Verhulst S. (2013). Telomere length reflects phenotypic quality and costs of reproduction in a long-lived seabird. Proc. R. Soc. Lond..

[71] Yip S.H., Sham P.C., Wang J. (2018). Evaluation of tools for highly variable gene discovery from single-cell RNA-seq data evaluation of tools for highly variable gene discovery from single-cell RNA-seq data. Brief. Bioinform..

[72] Cusanelli E., Angelica C., Romero P., Chartrand P. (2013). Telomeric noncoding RNA TERRA is induced by telomere shortening to nucleate telomerase molecules at short telomeres. Mol. Cell.

[73] Okamoto K., Iwano T., Tachibana M., Shinkai Y. (2008). Distinct roles of TRF1 in the regulation of telomere structure and lengthening. J. Biol. Chem..

[74] Kolquist K.A., Ellisen L.W., Counter C.M., Meyerson M., Tan L.K., Weinberg R.A., Haber D.A., Gerald W.L. (1998). Expression of TERT in early premalignant lesions and a subset cells in normal tissues. Nat. Genet..

[75] Ludlow A.T., Spangenburg E.E., Chin E.R., Cheng W.H., Roth S.M. (2014). Telomeres shorten in response to oxidative stress in mouse skeletal muscle fibers. J. Gerontol. Biol. Sci. Med. Sci. J..

[76] Schmutz I., de Lange T. (2016). Shelterin. Curr. Biol..

[77] Bejarano L., Schuhmacher A.J., Squatrito M., Blasco M.A. (2017). Inhibition of TRF1 telomere protein impairs tumor initiation and progression in glioblastoma mouse models and patient-derived xenografts. Cancer Cell.

[78] (2020). RGD (Rat Genome Database). (N.D). Telomere Maintenance 2 Gene. https://www.rgd.mcw.edu.

[79] Scheibe M., Arnoult N., Kappei D., Bucholz F., Decottignies A., Butter F., Mann M. (2013). Quantitative interaction screen of telomeric repeat-containing RNA reveal novel TERRA regulators. Genome Res..

[80] Maicher A., Kastner L., Dees M., Luke B. (2012). Deregulated telomere transcription causes replication—Dependent telomere shortening and promotes cellular senescence. Nucleic Acid Res..

[81] Prowse K.R., Greider C.W. (1995). Development and tissue specific regulation of mouse telomerase and telomere length. Proc. Natl. Acad. Sci. USA.

[82] Shay J.W., Bacchetti S. (1997). A survey of telomerase activity in human cancer. Eur. J. Cancer.

[83] Goichberg P., Chang J., Liao R., Leri A. (2014). Cardiac stem cells: Biology and clinical applications. Antioxid. Redox Signal..

[84] Bettin N., Pegorar C.O., Cusanelli E. (2019). The emerging roles of TERRA in telomere maintenance and genome stability. Cells.

[85] Yifan C., Tianzheng Y. (2021). Mouse liver is more resistant than skeletal muscle to heat-induced apoptosis. Cell Stress Chaperones.

[86] Lu A.L., Li X., Gu Y., Wright P.M., Chang D.Y. (2001). Repair of oxidative DNA damage:mechanisms and functions. Cell Biochem. Biophys..

[87] Marnett L.J. (2000). Oxyradicals and DNA damage. Carcinogenesis.

